# Impact of Digital Panoramic Radiograph Magnification on Vertical Measurement Accuracy

**DOI:** 10.1155/2015/452413

**Published:** 2015-10-19

**Authors:** Marc El Hage, Jean-Pierre Bernard, Christophe Combescure, Lydia Vazquez

**Affiliations:** ^1^Oral Surgery and Implantology Unit, Division of Oral and Maxillofacial Surgery, Department of Surgery, Faculty of Medicine, Geneva University Hospitals, rue Barthélemy-Menn 19, 1205 Geneva, Switzerland; ^2^Ardentis Clinique Dentaire Lausanne SA, Swiss Dental Clinics Group, voie du Chariot 6, 1003 Lausanne, Switzerland; ^3^Department of Oral Surgery, Oral Medicine, Oral and Maxillofacial Radiology, University Clinics of Dental Medicine, University of Geneva, rue Barthélemy-Menn 19, 1205 Geneva, Switzerland; ^4^Department of Health and Community Medicine, Center of Clinical Research and Division of Clinical Epidemiology, Faculty of Medicine, Geneva University Hospitals, rue Barthélemy-Menn 19, 1205 Geneva, Switzerland; ^5^Department of Orofacial Rehabilitation, Oral and Maxillofacial Radiology, University Clinics of Dental Medicine, University of Geneva, rue Barthélemy-Menn 19, 1205 Geneva, Switzerland

## Abstract

*Objectives.* The purpose of this panoramic radiography study was to assess the impact of image magnification on the accuracy of vertical measurements in the posterior mandible. *Methods.* Six dental implants, inserted in the posterior segments of a resin model, were used as reference objects. Two observers performed implant length measurements using a proprietary viewer with two preset image magnifications: the low (1.9 : 1) and the medium (3.4 : 1) image magnifications. They also measured the implant lengths in two Digital Imaging Communications in Medicine viewers set at low (1.9 : 1), medium (3.4 : 1), and high (10 : 1) image magnifications. *Results.* The error between the measured length and the real implant length was close to zero for all three viewers and image magnifications. The percentage of measurements equal to the real implant length was the highest (83.3%) for the high image magnification and below 30% for all viewers with the low image magnification. *Conclusions.* The high and medium image magnifications used in this study allowed accurate vertical measurements, with all three imaging programs, in the posterior segments of a mandibular model. This study suggests that a low image magnification should not be used for vertical measurements on digital panoramic radiographs when planning an implant in the posterior mandible.

## 1. Introduction

Panoramic radiography, a standard examination tool for planning of posterior mandibular dental implants, provides adequate radiographic evaluation with a low radiation dose [1–5]. In routine dental practice, proprietary dental imaging software programs as well as Digital Imaging Communications in Medicine (DICOM) viewers are used by clinicians for measurements on two-dimensional and three-dimensional images. Several authors have reported on the accuracy of linear measurements on cone beam computed tomography (CBCT) images [6–9]. Gaia et al. [9] used two imaging software programs to compare the precision and accuracy of linear measurements on CBCT images performed for Le Fort I osteotomy. Schulze et al. [10] examined the precision and accuracy of measurements on digital panoramic radiography under two image magnifications. The authors did the measurements with a single software program and concluded that high image magnification (2 : 1) lowered the measurement accuracy.

Clinicians use the image magnification setting on their computer screen which they perceive as being the most adapted for their daily practice. To the best of our knowledge, no studies on digital panoramic radiography have assessed, with different imaging software programs, the impact of image magnification on the accuracy of on-screen linear vertical measurements in the posterior mandible.

The purpose of this* in vitro* digital panoramic radiography study was to assess, with three imaging software programs, the impact of image magnification on the accuracy of vertical measurements in the posterior mandible.

## 2. Materials and Methods

### 2.1. Reference Object

**Figure 1:**
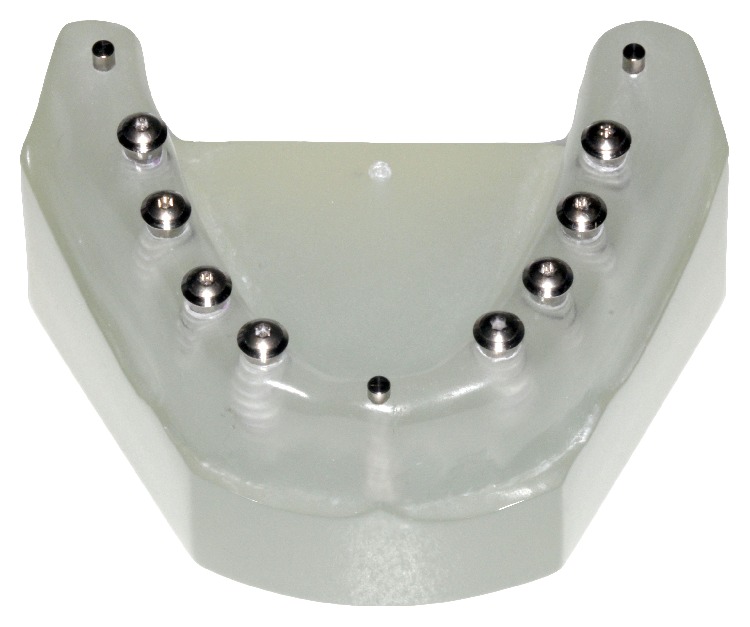
The custom-made resin model.

Eight standard diameter (4.1 mm) regular neck Straumann dental implants (Straumann AG, Basel, Switzerland) were inserted bilaterally in a radiopaque custom-made resin model, duplicated from a Straumann mandibular model, into sites corresponding to the canine, first and second premolar, and first molar positions. The implants were used as reference objects and their length was 8 mm or 10 mm alternatively (Figure 1).

### 2.2. Radiographic Examination

The panoramic radiographic examination was performed in a digital panoramic unit (CS 9300C, Carestream Health, Rochester, NY, USA). Exposure parameters were set at 60 kV, 2.5 mA, and a 180° rotation (14.3 s scan).

### 2.3. Measurements

Implants in the canine position were excluded, as this* in vitro* study required only implants in the posterior segments. Two trained oral surgeons, experienced in interpreting panoramic radiographs, did all the measurements. The 6 implants were measured twice randomly, 1 week apart, by the two observers on 3 imaging software programs: proprietary Kodak Dental Imaging Software (KDIS) (version 6.12.32.0; Kodak Dental Systems, Carestream Health, Rochester, NY, USA), open-source DICOM viewer OsiriX (version 1.2 64-bit; Pixmeo, Geneva, Switzerland), and web-based DICOM viewer Weasis (version 1.2.5; Weasis Team, Geneva, Switzerland).

### 2.4. Radiological Implant Length Evaluation

Each observer measured twice randomly the radiological length of the 6 implants in the posterior mandibular region. For the vertical measurements, both image magnifications preset by the KDIS proprietary viewer were used and were labeled K-medium (3.4 : 1) and K-low (1.9 : 1). With Weasis and OsiriX viewers, three image magnifications were used. W-medium and O-medium were equal to K-medium (3.4 : 1), W-low and O-low were equal to K-low (1.9 : 1), and a higher image magnification (labeled W-high and O-high) was set to 10 : 1. For each magnification setting, 24 measurements were performed.

### 2.5. Statistical Analysis

The error for each measurement (measured value minus real implant length) was assessed and its distribution was graphically represented by box plot and described by mean, standard deviation, and minimal and maximal values. The percentage of measurements with a null error was reported and compared between magnification settings with McNemar's test. The interobserver and intraobserver agreements were analyzed using Bland and Altman analysis. Mean and standard deviation of error (between observers and between sessions) and limits of agreement were reported. The maximum tolerated difference was set at 0.3 mm. Standard deviations compared the magnification settings with a Morgan-Pitman procedure [11, 12]. Statistical analysis was performed using S-PLUS 8.0 (Insightful Corp., Seattle, WA).

Implant length values measured in Weasis and OsiriX viewers were rounded to the nearest tenth for comparison with the values measured in KDIS viewer as the authors of the present study agreed that a precision of more than a tenth of a millimeter was clinically irrelevant in dental implant planning. Statistical analysis confirmed that results were identical for rounded and nonrounded values.

## 3. Results

### 3.1. Error Description

**Figure 2:**
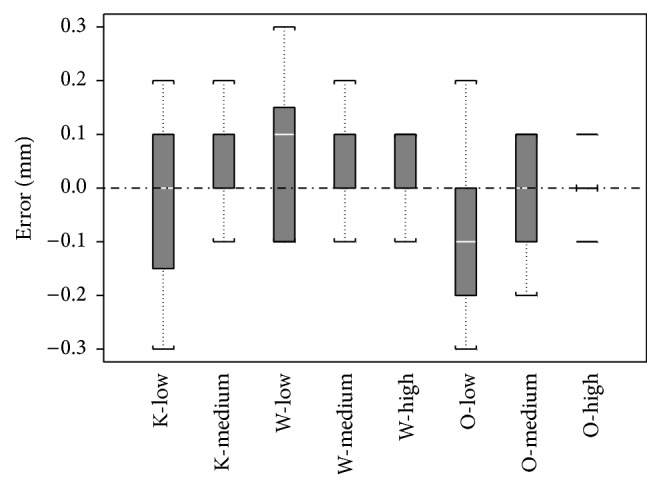
Distribution of the error in mm (box plots). The white horizontal line represents the median, the grey rectangle represents the interquartile range, and the lower and upper limits are the minimums and maximums.

The error between the measured length and the real implant length was close to zero for all three viewers and image magnifications (Table 1): the mean errors ranged from −0.13 mm (O-low) to +0.05 mm (W-low). Distribution of errors is shown in Figure 2. The percentage of measurements equal to the real implant length was the highest for O-high (83.3%) and below 30% for all viewers with the low image magnification. Globally, this percentage increased significantly with magnification of the image (McNemar, O-high versus O-medium: *χ* ^2^ = 7.56, df = 1, and *p* = 0.006; O-high versus O-low: *χ* ^2^ = 11.08, df = 1, and *p* = 0.0009; K-low versus K-medium: *χ* ^2^ = 5.82, df = 1, and *p* = 0.02; W-medium versus W-low: *χ* ^2^ = 6.75, df = 1, and *p* = 0.009).

### 3.2. Interobserver and Intraobserver Agreements

**Figure 3:**
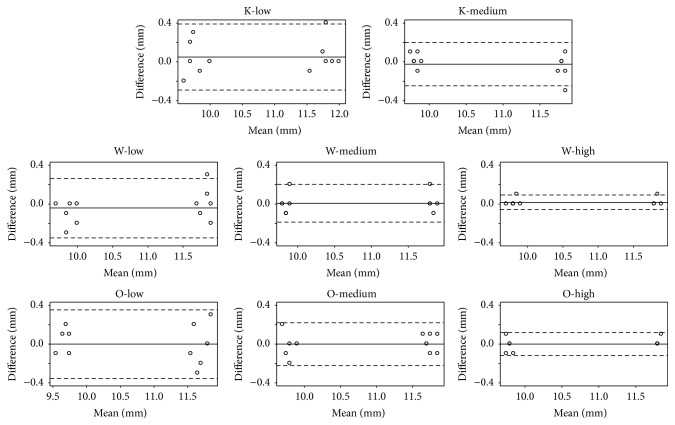
Intraobserver reproducibility (Bland and Altman plots). The straight horizontal line represents the mean difference between sessions and the dashed horizontal lines represent the limits of agreements.

The difference between values measured during both sessions (intraobserver difference) was analyzed. The mean difference was close to zero for all viewers and image magnifications (Table 2). The Bland and Altman plots are represented in Figure 3. For all viewers, the limits of agreement were smaller than ±0.30 mm with the medium or high image magnifications but not with the low image magnification. Intraobserver differences were closer to zero with the high image magnification compared to those with the low image magnification (Morgan-Pitman, W: *t* = 7.27, df = 10, and *p* < 0.0001; O: *t* = 4.23, df = 10, and *p* = 0.002). Intraobserver differences were also closer to zero with the high image magnification compared to those with the medium image magnification (Morgan-Pitman, W: *t* = 3.80, df = 10, and *p* = 0.004; O: *t* = 2.50, df = 10, and *p* = 0.03). No difference was detected between low and medium magnification settings.

**Figure 4:**
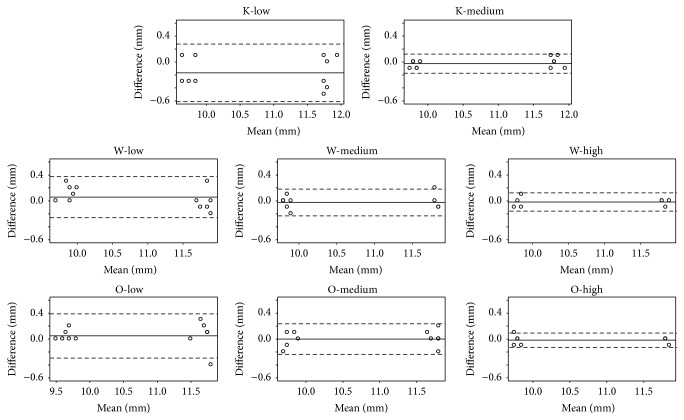
Interobserver reproducibility (Bland and Altman plots). The straight horizontal line represents the mean difference between observers and the dashed horizontal lines represent the limits of agreements.

The Bland and Altman plots representing the differences between observers (interobserver differences) are shown in Figure 4. The interpretation of the interobserver agreement was similar to the intraobserver agreement.

## 4. Discussion

Digital radiographic imaging systems have become widely available, allowing the use of digital panoramic radiographs for dental implant treatments. This quick, simple, low-cost, and low-dose diagnostic tool allows evaluation of the available bone height before placement of posterior mandibular implants [4, 5]. A safety margin of at least 2 mm between the implant's tip and the mandibular canal is recommended [4]. Proprietary software-based measurements tools are commonly used for vertical measurements in premolar and molar mandibular segments on digital panoramic radiographs [13–17]. Precision and accuracy of measurements in digital panoramic radiography using a single measurement software program have been examined under two magnification settings [10]. To our knowledge, no digital panoramic radiography studies have assessed impact of image magnification on the accuracy of measurements using various imaging software programs, including DICOM viewers. Yet, DICOM and other software programs have been used to examine the accuracy of CBCT measurements [6, 8, 9, 18]. Gaia et al. [9] compared linear measurements in CBCT images using Vitrea 3.8.1 (Vital Images Inc., Plymouth, MN) viewer and the same open-source DICOM viewer OsiriX 1.2 64-bit (Pixmeo, Geneva, Switzerland) as the one used in the present* in vitro* study. Vitrea software showed measurements closer to the dry skull values (gold standard) compared to measurements obtained with OsiriX [9]. The same authors concluded that measurements with Vitrea were precise and accurate, whereas the OsiriX viewer was not successful in producing accurate linear measurements for Le Fort I osteotomy. In the present study, the high image magnification (10 : 1) used to measure reference objects might explain why our results do not support the conclusions of Gaia et al. [9] regarding the precision of OsiriX viewer. Furthermore, OsiriX software has been used in another study to evaluate the accuracy and reliability of measurements on 3D computed tomography images of pig femur [19]. Differences between real and OsiriX measurements were less than 0.1 mm and the authors concluded that OsiriX software was very reliable for measurements purposes. In the present study, measurements performed with OsiriX under the high image magnification (O-high) showed the lowest error value. In addition, the percentage of measurements equal to the real implant length was the highest for O-high (83.3%). Furthermore, the present study demonstrated that there were no statistically significant differences between the three software programs (proprietary and DICOM viewers) when measuring vertical linear distances on panoramic radiographs. Our results corroborate other studies using various proprietary measurement software programs on digital panoramic radiographs in premolar and molar mandibular segments [15–17, 20].

Agreements between observers and between measurement sessions appeared to be optimal when a high (10 : 1) image magnification was used for on-screen vertical measurements in the posterior segments of a mandibular model. Standard deviations were significantly lower with the high image magnification compared to those with the low image magnification. The high and medium image magnifications used in this* in vitro* study allowed accurate vertical measurements with all three imaging programs. Our results contradict Schulze et al. [10] reporting that measurements with a high image magnification (2 : 1) were less accurate than those with a lower magnification (1 : 1); the authors recommended that digital measurements should not be performed on magnified images. The same authors mention the reason for this; the pixel size was a limiting factor for larger magnifications since high magnification resulted in a relatively diffuse image on the screen, which made it increasingly difficult to identify the borders of any object shown on the screen. In the present study, the observers were not confronted with such a problem, and this is a result of high initial image resolution and small pixel size, features that are common to the current panoramic digital image acquisition systems and imaging software programs.

Certain limitations do exist in the present study. This study was limited to measurements on 8 mm and 10 mm implants which may have underpowered measurement inaccuracies. When analyzing the percentages of measurements with a null error, a higher image magnification globally improved the accuracy. With the sample size of our study, the statistical power was 80% to detect a difference around 30% in the proportion of measurements without error between two image magnifications, and certain specific differences below 10% (such as the difference between O-low and O-medium) were not statistically significant. Furthermore, the single panoramic acquisition of parallel mandibular implants placed in the center of the resin crest may have positively impacted the measurements. Moreover, another limitation was the fact that a static model was used for measurements. Susceptibility to patient movement during acquisition and error of head positioning were not analyzed, knowing that these factors could affect the measurements [21]. Our results nevertheless showed good measurement accuracy on a static model with three imaging programs and three image magnifications. Results might also have been positively impacted by the observers' experience in interpreting panoramic images, whereas clinicians with different experiences in measurements on a screen would have been better representative of routine clinical practice.

## 5. Conclusion

The high and medium image magnifications used in this* in vitro* study allowed, with all three imaging programs, accurate vertical measurements in the posterior segments of a mandibular model. This study suggests that a low image magnification should not be used for vertical measurements on digital panoramic radiographs when planning an implant in the posterior mandible.

## Figures and Tables

**Table 1:** Measurement error description. SD: standard deviation.

	Magnification	Mean error in mm (SD)	Median error in mm [min-max]	% of measurements with a null error
K-low	1.9 : 1	−0.02 (0.16)	0.00 [−0.30; 0.20]	5/24 (20.8%)
K-medium	3.4 : 1	0.02 (0.07)	0.00 [−0.10; 0.20]	14/24 (58.3%)
W-low	1.9 : 1	0.05 (0.13)	0.10 [−0.10; 0.30]	4/24 (16.7%)
W-medium	3.4 : 1	0.04 (0.06)	0.00 [−0.10; 0.20]	14/24 (58.3%)
W-high	10 : 1	0.03 (0.06)	0.00 [−0.10; 0.10]	14/24 (58.3%)
O-low	1.9 : 1	−0.13 (0.13)	−0.10 [−0.30; 0.20]	7/24 (29.2%)
O-medium	3.4 : 1	−0.02 (0.10)	0.00 [−0.20; 0.10]	8/24 (33.3%)
O-high	10 : 1	0.00 (0.04)	0.00 [−0.10; 0.10]	20/24 (83.3%)

**Table 2:** Intraobserver and interobserver differences and limits of agreement. SD: standard deviation.

	Difference (mm)		Limits of agreement (mm)	
	Mean (SD)	Median [min-max]	Lower	Upper
Intraobserver difference				
K-low	0.05 (0.17)	0.0 [−0.2; 0.4]	−0.29	0.39
K-medium	−0.03 (0.11)	0.0 [−0.3; 0.1]	−0.25	0.20
W-low	−0.04 (0.16)	0.0 [−0.3; 0.3]	−0.35	0.26
W-medium	0.01 (0.10)	0.0 [−0.1; 0.2]	−0.19	0.20
W-high	0.02 (0.04)	0.0 [0.0; 0.1]	−0.06	0.09
O-low	0.00 (0.18)	−0.05 [−0.3; 0.3]	−0.35	0.35
O-medium	0.00 (0.11)	0.0 [−0.2, 0.2]	−0.22	0.22
O-high	0.00 (0.06)	0.0 [−0.1, 0.1]	−0.12	0.12
Interobserver difference				
K-low	−0.17 (0.23)	−0.3 [−0.5; 0.1]	−0.61	0.28
K-medium	−0.03 (0.08)	0.0 [−0.1; 0.1]	−0.17	0.12
W-low	0.06 (0.16)	0.0 [−0.2; 0.3]	−0.26	0.38
W-medium	−0.03 (0.11)	0.0 [−0.2, 0.2]	−0.23	0.18
W-high	−0.02 (0.07)	0.0 [−0.1, 0.1]	−0.16	0.12
O-low	0.05 (0.17)	0.05 [−0.4; 0.3]	−0.29	0.39
O-medium	0.00 (0.12)	0.0 [−0.2; 0.2]	−0.24	0.24
O-high	−0.02 (0.06)	0.0 [−0.1; 0.1]	−0.13	0.10
